# Beyond research: improved perinatal care through scale-up of a Moyo fetal heart rate monitor coupled with simulation training in northern Tanzania for helping babies breathe

**DOI:** 10.1186/s12887-022-03249-7

**Published:** 2022-04-11

**Authors:** Yuda Munyaw, Joshua Gidabayda, Anita Yeconia, Godfrey Guga, Esto Mduma, Paschal Mdoe

**Affiliations:** 1 Department of Obstetrics and Gynecology, Haydom Lutheran Hospital, P.O BOX 9000, Haydom, Mbulu Tanzania; 2 Department of Pediatrics, Haydom Lutheran Hospital, P.O BOX 9000, Haydom, Mbulu Tanzania; 3 Research Centre, Haydom Lutheran Hospital, P.O BOX 9000, Haydom, Mbulu Tanzania

**Keywords:** Fetal heart rate, Moyo, Helping babies breathe, Scale-up, Perinatal deaths

## Abstract

**Background:**

The purpose of this project was to improve perinatal survival by introducing Moyo Fetal Heart Rate (FHR) Monitor coupled with neonatal resuscitation simulation training.

**Methods:**

The implementation was done at three district hospitals. We assessed health care workers’ (HCW’s) skills and perinatal death trends during implementation. Baseline data were collected from the hospitals before implementation. Newborn resuscitation (NR) skills were assessed before and after simulation training. Assessment of perinatal outcomes was done over 2 years of implementation. We used descriptive analysis; a t-test (paired and independent two-sample) and a one-way Anova test to report the findings.

**Results:**

A total of 107 HCW’s were trained on FHR monitoring using Moyo and NR knowledge and skills using NeoNatalie simulators. The knowledge increased post-training by 13.6% (*p* <  0.001). Skills score was increased by 25.5 and 38.2% for OSCE A and B respectively (*p* <  0.001). The overall fresh stillbirths rate dropped from 9 to 5 deaths per 1000 total births and early neonatal deaths at 7 days from 5 to 3 (*p* <  0.05) deaths per 1000 live births over 2 years of implementation.

**Conclusion:**

There was a significant improvement of newborn resuscitation skills among HCW’s and neonatal survival at 2 years. Newborn resuscitation training coupling with Moyo FHR monitor has shown potential for improving perinatal survival. However, further evaluation is needed to explore the full potential of the package.

## Background

Perinatal deaths refer to a combination of fetal deaths (after 28 weeks) and live births with only brief survival (1 week) and are grouped on the assumption that similar factors are associated with these losses [1]. The majority of perinatal deaths occur in low-resource settings. A Meta-analysis study of demographic health surveys showed a perinatal mortality rate (PMR) of 34.7 across 21 countries in sub-Saharan Africa. In this analysis, the highest PMR was observed in Tanzania (39.5) [2].

In a hospital-based survey in Tanzania (2006–2015), neonatal mortality rates were reported to be increasing from 2.6 to 10.4 deaths per 1000 live births. Early neonates contributed 90% to these death rates constantly over time. In this survey, the leading cause of early neonatal deaths was birth asphyxia [3]. A facility-based case-control study in Zanzibar reported 59 stillbirths per 1000 total births, in which 52% had fetal heart rate (FHR) at admission [4].

Poor quality of intrapartum care contributes to perinatal mortality. An increased risk of intrapartum stillbirth when FHR is inadequately monitored is evident [5]. It is further reported that a shortage of FHR measurement devices is a barrier to FHR monitoring standards [6]. For the FHR monitoring to be effective, the device used must be reliable and convenient for both the HCW’ and pregnant women in labor. In high resource settings, FHR during labor is mostly monitored continuously using cardiotocography (CTG). In Tanzania, FHR is monitored intermittently mostly using Pinard fetoscope at lower health facilities or rarely by handheld Dopplers.

Doppler fetal monitors are becoming widely available and used in clinical practice in low-resource settings. These monitors use ultrasound technology to detect fetal heartbeats as early as 12 weeks [7]. One advantage of the Doppler fetal monitor is the electronic audio output, which allows users and laboring women to hear the fetal heartbeat. However, the dopplers carry some disadvantages including high cost, source of costly power (battery or electricity), and the need for technical maintenance. With further technological development, Doppler remains an important tool for the assessment of fetal well-being globally [7].

A recent systematic review study has shown that Doppler is superior at detecting abnormal intrapartum FHR compared to Pinard [8]. This review further shows that using Doppler on admission helps to accurately measure fetal deaths occurring after facility admission [8]. Trials conducted at referral teaching hospitals in Tanzania comparing the effectiveness of a novel Doppler FHR monitor (Moyo) compared with Pinard and classic Doppler have shown that Moyo is effective in detecting abnormal FHR [9, 10].

Moyo is a strap-on automatic multicrystal Doppler device (named Moyo, Laerdal Global Health) that has been recently introduced into clinical practice. Moyo is designed for both intermittent and continuous FHR monitoring. With a 9-crystal sensor, Moyo can accurately detect FHR within 5 s and can differentiate maternal and fetal heart rates. Moyo has 30 min histogram display of FHR trends which can be reviewed by the HCW to assess FHR. The device has an inbuilt audio-visual alarm if abnormal FHR is detected. Moyo is lightweight and portable and thus can allow a laboring woman to move around and choose the birth position.

Apart from the use of innovative devices, strengthening the knowledge and skills of health care workers (HCWs) to provide neonatal resuscitation (NR) is critical to reducing perinatal morbidity and mortality. American Academy of Pediatrics and its global partners created the Helping Babies Breathe (HBB) program, which teaches basic NR techniques to birth attendants in low-resource settings (LRSs).

Systematic reviews have demonstrated significant improvement of HCW’s knowledge and skills after HBB simulation training [11]. However, it has been observed generally that the knowledge and skills gained fall off after initial training [12]. This is a barrier to HBB’s success. To improve skills retention among HCWs, several different HBB training approaches have been recommended [13–15].

The impact of HBB on perinatal outcomes has been evaluated with conflicting results. A pilot HBB study in eight hospitals in Tanzania between 2009 and 2011 resulted in a 47% reduction in hospital-based neonatal mortality within 24 h [16]. Several other studies in resource-limited settings have reported a positive impact of HBB training on intrapartum and early neonatal deaths. A study by KC et al. showed a change in intrapartum-related stillbirths from 9.0 to 3.2/1000 births after HBB implementation [17]. A systematic review study further showed the strongest decline in intrapartum-related stillbirths and one-day newborn deaths after HBB training [18]. However, frequent HBB training is necessary to sustain the impact on mortality. Mduma et al. showed significantly improved perinatal survival over 6 years of implementing low dose high frequency (LDHF) training of HBB [19].

Most countries in LRSs including Tanzania are far away from the recommended Sustainable Development Goals (SDGs) targets of 12 stillbirths per 1000 total births and 12 neonatal deaths per 1000 livebirths [20]. To reduce these deaths, there is an urgent need to develop and introduce tools/technologies and scale up these innovations to reach lower-level health facilities.

Beyond research to perinatal care improvement project was implemented to introduce Moyo Doppler device for FHR monitoring coupled with simulation HBB training at three district hospitals in northern Tanzania. This study aimed to assess the contribution of the project on HCW’s HBB skills and perinatal mortality over 2 years of implementation.

## Materials and methods

Beyond research to perinatal care improvement project was implemented at three district hospitals namely Mbulu, Babati, and Hanang in northern Tanzania. The rate of perinatal mortality in northern Tanzania was 32 per 1000 pregnancies as per the last Demographic Health Survey (DHS) [21].

The district hospitals provide comprehensive emergency obstetric and newborn care services. They attend pregnant women direct from home and as referrals from dispensaries and health centers in the surrounding area. They refer complicated cases of pregnant women and newborns to referral hospitals in the surrounding.

Haydom Lutheran Hospital was involved in the national HBB pilot project 2009, national HBB rollout program 2013–2015, and Moyo innovation (Safer Births sub-project) 2017. The hospital benefited in terms of staff capacity building including master trainers in HBB and FHR monitoring using Moyo Doppler. This project technical team comprised of an obstetrician, pediatrician, and four Moyo and HBB master trainer midwives experienced in labor care practices and use of Moyo devices.

The master trainers conducted a trainer of trainees (TOTs) course, four participants from each district hospital were involved. The TOTs training lasted for 2 days at HLH (1 day for HBB and 1 day for Moyo). The approach was hands-on, using a NeoNatalie simulator and upright bag-mask ventilator for HBB training. We further trained them on the use of Moyo for FHR monitoring and interpretation.

TOTs training was followed by training nurses/midwives and doctors working at maternity and theatre units of respective hospitals. The training was done at each district hospital setting by master trainers together with TOTs. HBB knowledge and skills for each participant were assessed by administering knowledge check questions and objective structured clinical evaluations (OSCE). This was done before and after training sessions. We used knowledge check questions, OSCE A and B available in HBB educational materials. We decided to use these evaluation tools because they have been validated and used elsewhere and they can be reproduced. Evaluations were supervised by the same master trainers in all training sessions.

Skills trained and evaluated include newborn assessment, drying, suction, stimulation, and bag-mask ventilation. We used curriculum and standard educational materials for HBB training as developed by the American Academy of Pediatrics (Second edition, English version). Participants were further trained on FHR monitoring, intermittent and continuous using the Moyo device. We used local guidelines that FHR to be reviewed every 30 min in the first stage and every 15 min in the second stage of labor and interpreted as normal when the rate is 110–160 and abnormal when the rate is below 110 or above 160 beats per minute.

After the training, facilities were provided with Moyo Doppler devices, NeoNataliae simulators, and upright bag-mask ventilators for self-practice and clinical care. Laerdal Foundation supported the project with training materials-Moyo Doppler, NeoNataliae simulators, upright bag-mask ventilators, and Penguin suction. Apart from the TOTs supervising ongoing practices at the hospitals to maintain and sustain skills, there were no other quality improvement activities related to maternal and newborn health at the district hospitals.

We hypothesized that the HBB skills score of the HCW’s at district hospitals before the training was 65%. We planned to increase the skills score to 90% after the training. To achieve that we needed to train 102 HCW’s in all three district hospitals. To increase the strength of the study we added 20%, therefore 120 HCW’s were planned to be trained.

Training data was obtained by recording test scores before and after training sessions. To obtain data on clinical outcome assessment, we collected baseline information from the district hospitals for the year 2017 before we implemented the project in 2018 and 2019. Data on total deliveries, live births, fresh stillbirths, and early neonatal deaths were gathered from each hospital. After 2 years of implementation, we collected the same clinical data from the same source. The main source of clinical outcomes data was District Health Information Software (DHIS2). The DHIS2 compiles and presents electronic routine health services information collected through the Health Management Information System.

Information from DHIS2 was collected using a structured data collection form. To ensure data quality, validation of data was done by an investigator in cooperation with data focal persons at the district hospitals. Double data entry was performed by the data clerk and discrepancies were resolved.

Data analysis was performed using R 3.6.2(Foundation for Statistical Computing, Vienna, Austria). We used descriptive analysis (mean, standard deviation, count, and percent), paired t-test was used to assess the significant differences between the overall mean scores of pre-tests and post-test of each skills question and knowledge. An Independent two-sample t-test was used to assess the significant differences of skills gained among the binary demographic variables. One-way analysis of variance was used to assess the comparison of skills and knowledge gain among the demographic variable with multiple levels and significant differences between perinatal outcomes among the years before and during implementation. Tukey honest significant differences were used as a post hoc test for comparing significant differences between the means of the levels of a factor. The level of significance was set at 0.05.

## Results

A total of 107 HCWs were trained on FHR monitoring using Moyo Doppler and HBB using NeoNatalie simulators. The distribution of HCWs was highest in Babati hospital (40%) and lowest in Mbulu hospital (27%). More than two-thirds were female and about three quarter were nurses. About 60% had age below 40 years and half of them had less than 10 years of working experience (Table 1).

**Table 1:** Demographic characteristics of health care workers trained

Variable	Total ***N*** = 107	Babati ***N*** = 43	Hanang ***N*** = 35	Mbulu ***N*** = 29
**Gender**				
Male	32 (30)	10 (23)	11 (31)	11 (38)
Female	75 (70)	33 (77)	24 (69)	18 (62)
**CADRE**				
Doctor	29 (27)	11 (26)	10 (29)	8 (28)
Nurse	78 (73)	32 (74)	25 (71)	21 (72)
**Age**				
< 30 yrs	21 (20)	6 (14)	8 (23)	7 (24)
30-39 yrs	44 (41)	16 (37)	17 (49)	11 (38)
40-49 yrs	28 (26)	13 (30)	8 (23)	7 (24)
50 yrs. +	14 (13)	8 (19)	2 (6)	4 (14)
**Working experience**				
< 10 yrs	55 (51)	19 (44)	20 (57)	16 (55)
10-19 yrs	36 (34)	15 (35)	13 (37)	8 (28)
20 yrs. +	16 (15)	9 (21)	2 (6)	5 (17)

HBB skills were assessed using OSCE tests pre-and post-training. Skill gain was 25.5 and 38.2% for OSCE A and B respectively with significant differences between pre-test and post-test scores. Significant differences in skills gained were only observed between the sites in OSCE B, where Hanang was observed to have a high level of skills gained about 50.9% and Babati was observed to have a low level of skills gain of 31.6% (Table 2).

**Table 2:** HBB skills and knowledge evaluation scores

	Pre-score (%) – Mean (SD)	Post-score (%) – Mean (SD)	Skills gain – Mean (SD)	***P***-value
**OSCE A**				
**Skills**	58.98 (24.61)	84.22 (18.92)	25.24 (24.66)	< 0.001
**Site**				0.119
Babati	70.73 (16.99)	89.83 (14.85)	19.10 (20.35)	
Hanang	57.38 (24.82)	87.14 (15.96)	29.76 (25.99)	
Mbulu	43.21 (25.43)	71.92 (22.67)	28.71 (27.68)	
**Gender**				0.949
Female	60.42 (23.39)	85.76 (16.92)	25.34 (25.31)	
Male	55.65 (27.34)	80.65 (22.82)	25.00 (23.48)	
**Age group**				0.521
< 30 yrs	59.52 (23.02)	82.54 (16.86)	23.02 (27.12)	
30 - 39 yrs	59.96 (24.67)	83.94 (18.67)	23.98 (24.52)	
40 - 49 yrs	59.88 (26.05)	91.05 (11.77)	31.17 (25.71)	
50 yrs. +	53.57 (25.89)	74.40 (28.59)	20.83 (19.00)	
**Working experiences**				0.779
< 10 yrs	59.75 (24.23)	83.65 (18.78)	23.90 (25.59)	
10-19 yrs	61.27 (23.47)	88.97 (12.43)	27.70 (24.77)	
20 yrs. +	51.56 (28.26)	76.04 (27.37)	24.47 (22.26)	
**Cadre**				0.5921
Doctors	56.85 (23.79)	84.23 (20.58)	27.39 (23.23)	
Nurses	59.78 (25.01)	84.22 (18.41)	24.44 (25.28)	
**OSCE B**				
**Skills**	39.93 (27.05)	78.10 (11.96)	38.17 (26.40)	< 0.001
**Site**				0.0016
Babati	48.17 (28.24)	79.73 (12.56)	31.56 (27.45)	
Hanang	27.50 (23.30)	78.40 (8.21)	50.90 (22.13)	
Mbulu	43.52 (24.61)	75.23 (14.76)	31.71 (24.57)	
**Gender**				0.9324
Female	40.80 (27.95)	78.82 (10.68)	38.02 (25.06)	
Male	37.90 (25.15)	76.41 (14.59)	38.51 (29.72)	
**Age group**				0.481
< 30 yrs	42.86 (17.15)	75.60 (15.92)	32.74 (19.76)	
30 - 39 yrs	37.96 (26.97)	80.95 (9.37)	42.99 (24.37)	
40 - 49 yrs	41.44 (30.82)	77.55 (12.29)	36.11 (32.02)	
50 yrs. +	38.39 (33.42)	74.56 (10.54)	36.16 (29.23)	
**Working experiences**				0.178
< 10 yrs	40.45 (23.31)	78.54 (12.59)	38.09 (23.30)	
10-19 yrs	37.50 (29.48)	80.52 (8.94)	43.02 (25.60)	
20 yrs. +	43.36 (33.96)	71.49 (13.69)	28.13 (35.43)	
**Cadre**				0.0518
Doctors	28.35 (23.72)	74.78 (11.47)	46.43 (29.34)	
Nurses	44.25 (27.08)	79.34 (11.98)	35.09 (24.72)	
**Knowledge**	75.0 (16.7)	88.7 (13.3)	13.6 (19.4)	< 0.001
**Site**				0.567
Babati	70.4 (19.5)	86.3 (18.1)	15.9 (26.9)	
Hanang	78.4 (13.1)	88.7 (9.7)	10.3 (9.5)	
Mbulu	77.3 (15.5)	91.3 (8.7)	13.9 (15.6)	

An average score of knowledge gain was 13.6%, with a significant difference between pre-test score and post-test score, where the highest average score of knowledge gain was observed Babati with 15.9% and Hanang had the lowest score gained 10.3% with no significant difference between sites (Table 2).

An overall FSB rate was around 9 cases per 1000 births in 2017 and dropped to 5 cases per 1000 births in 2019, where the rates vary from site to site. The early neonatal death rate within 24 h and 7 days has dropped from 4 cases and 5 cases per 1000 live births respectively in 2017 to 2 cases and 3 cases per 1000 live births in 2019 (Table 3).

**Table 3:** Perinatal data before (2017) and during implementation (2018–2019)

	Years	Site			Total	Overall rate (per 1000)
		Babati	Hanang	Mbulu		
**Total births**	2017	3659	2849	3747	10,255	–
	2018	3352	2784	3795	9931	–
	2019	3622	2964	3275	9861	–
**Livebirths**	2017	3587	2775	3669	10,031	–
	2018	3308	2701	3745	9754	–
	2019	3593	2928	3234	9755	–
**Total Stillbirths**	2017	72	74	78	224	22
	2018	44	83	50	177	18
	2019	29	36	41	106	11
**Fresh Stillbirths**	2017	14	36	42	92	9
	2018	9	40	21	70	8
	2019	6	18	19	43	5
**Early neonatal deaths within 24 h**	2017	11	14	14	39	4
	2018	7	9	13	29	3
	2019	5	7	5	17	2
**Early neonatal deaths within 7 days**	2017	17	15	18	50	5
	2018	11	11	20	42	5
	2019	9	8	12	29	3

The significant differences between the average rates of perinatal mortality among the years before and during the implementation were observed on early neonatal death at 24 h and 7 days, where the year 2019 had a lower early neonatal death rate compared to the year 2017 and their comparison is significant (Table 4).

**Table 4:** Analysis of variance of the perinatal outcome by years (2017–2019) with post hoc test

**Anova**					
Fresh stillbirths					
** Source**	**df**	**Sum Sq.**	**Mean Sq**	**F**	**P**
Years	2	33.56	16.78	0.766	0.506
Residuals	6	131.45	21.91		
Early neonatal death 24 h					
** Source**	**df**	**Sum Sq.**	**Mean Sq**	**F**	**P**
Years	2	7.28	3.64	6.067	0.0362
Residuals	6	3.60	0.60		
Early neonatal death 7 days					
** Source**	**df**	**Sum Sq.**	**Mean Sq**	**F**	**P**
Years	2	6.327	3.163	6.096	0.0359
Residuals	6	3.113	0.519		
**Tukey multiple comparisons of means - 95% family-wise confidence level**					
Fresh stillbirths					
** years**	**differences**	**LL**	**UL**	**P adj**	
2018–2017	−1.666667	−13.39291	10.059573	0.9021183	
2019–2017	−4.666667	−16.39291	7.059573	0.4845955	
2019–2018	−3.000000	−14.72624	8.726239	0.7251654	
Early neonatal death 24 h					
** years**	**differences**	**LL**	**UL**	**P adj**	
2018–2017	−1.0	−2.940547	0.9405471	0.3231132	
2019–2017	−2.2	−4.140547	−0.2594529	0.0304650	
2019–2018	−1.2	−3.140547	0.7405471	0.2195134	
Early neonatal death 7 days					
** years**	**differences**	**LL**	**UL**	**P adj**	
2018–2017	−0.7666667	− 2.571287	1.0379533	0.4436499	
2019–2017	−2.0333333	−3.837953	−0.2287134	0.0312455	
2019–2018	−1.2666667	−3.071287	0.5379533	0.1587973	

*LL* Lower Limit, *UL* Upper Limit

**Fig. 1:**
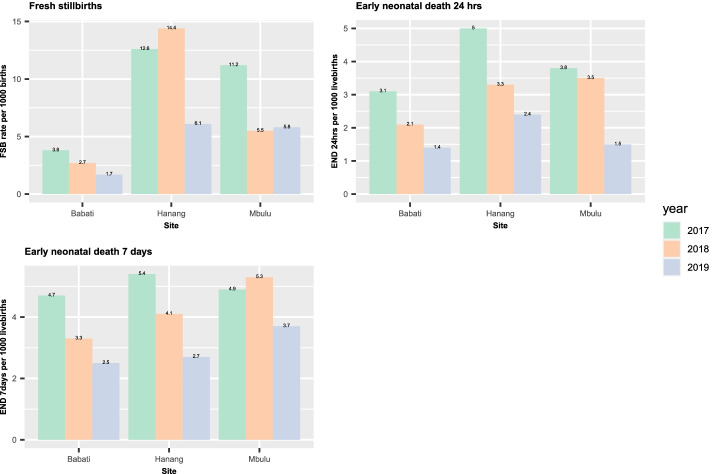
Perinatal deaths rates before (2017) and during implementation (2018–2019)

Generally, the perinatal outcomes (FSB and neonatal death within 7 days) seem to be decreasing over implementation years, though in some of the sites the decline is not smooth. For FSB, Hanang was observed to have high FSB in all years compared to other sites, whereas small differences were observed in early neonatal death rates at 7 days between the sites among all years (Fig. 1).

## Discussion

In this project, we were able to increase NR skills significantly through simulation training of HCWs and introduce novel FHR monitoring Doppler at district hospitals. The availability of essential medical devices and strategic training of HCWs is central to reducing perinatal morbidity and mortality. In LRSs, innovative medical devices are rarely available at lower health facilities, including the Moyo Doppler device for FHR monitoring. In this project, we rolled out this innovative device coupled with HBB training. Previous studies have demonstrated the effectiveness of this device in FHR monitoring [9, 10] and its acceptability among midwives and laboring women [22]. To our understanding, this is the first scale-up project to roll out an innovative novel FHR monitor (Moyo) coupled with HBB training at lower health facilities in Tanzania.

Training on maternal and newborn HCW’s is a crucial intervention to improve perinatal survival. This project has achieved significant HBB knowledge and skills gain after the training by 13 and 31% respectively. The difference of gain in knowledge and skills might be explained by the fact that the majority of participants were nurses from the labor ward who have experience with NR skills in their routine work hence easy to learn skills during the training.

This achievement is consistent with what has been reported in other studies underlining that HBB increased the acquisition rate of knowledge and skills of basic NR [11]. The nature of HBB simulation training – curriculum design, educational materials, and experienced master trainers may have contributed to the gained knowledge and skills. The acquired HBB knowledge and skills serve as important professional capacity building among involved health care workers to improve perinatal care.

The scale-up of Moyo coupled with HBB through this project was associated with improvement in clinical outcomes. The data indicates the reduction of stillbirths, 24 h and 7 days neonatal deaths. Previous studies using Moyo alone didn’t show a reduction in perinatal deaths as they were not coupled with newborn resuscitation training. In this study, FHRs of women who are in active labor was monitored using Moyo regardless of risk status. FHR monitoring followed the local guideline of measuring FHR after every 30 min for the first stage of labor and every 15 min for the second stage of labor for low-risk labors. For high-risk labors, Moyo was strapped on and FHR monitored continuously throughout.

Moyo Doppler has been reported to increase detection of abnormal FHR, which is a predictor of perinatal death. The risk of stillbirth is likely to decline when detection of abnormal FHR is coupled with recommended interventions such as intrauterine resuscitation or emergency delivery. The involved district hospitals are capable of providing emergency delivery services in case abnormal FHR is detected. Hence, proper utilization of the newly introduced device is probably going to further improve the birth outcomes.

Results of innovations in health services have a greater and more rapid impact on improving health when scaled up to reach facilities and communities. In addition, sustainability and local ownership are key principles of scale-up success [23]. In this project, local Trainers of Trainees (TOTs) were trained to support the use of Moyo for FHR monitoring and ongoing HBB practices at the facilities to sustain knowledge and skills. Materials for training and clinical care use were supplied to respective hospitals.

Addressing the perinatal death challenge by combining innovative tools (Moyo Doppler) and training modality (simulation) is the main strength of this project. Limitations include few HCWs with previous HBB training, this might have affected training skills scores. Some trained HCWs were shifted to other departments and some retired during implementation hence their gained knowledge and skills were not fully exploited. The results seen may have been affected by other unrecognized competing factors.

## Conclusion

Implementation of this project has resulted in an increased HBB knowledge and skills and is associated with an improvement in perinatal survival. An implementation approach combining Moyo and HBB at a large scale is recommended to further evaluate the impact on perinatal mortality.

## Data Availability

The datasets used during the current study are available from the corresponding author on reasonable request.

## Abbreviations

DHIS: District health information system
FHR: Fetal heart rate
FSB: Fresh stillbirth
HBB: Helping babies breathe
HCW: Health care workers
OSCE: Objective structured clinical evaluation
TOT: Trainer of trainees
NR: Newborn resuscitations
LRSs: Low resource settings
